# Solution‐Processed Semiconductor Materials as Cathode Interlayers for Organic Solar Cells

**DOI:** 10.1002/advs.202304673

**Published:** 2023-10-26

**Authors:** Yanhe Xiang, Bowei Xu, Ying Li

**Affiliations:** ^1^ State Key Laboratory of Chemical Resource Engineering College of Materials Science and Engineering Beijing University of Chemical Technology Beijing 100029 P. R. China; ^2^ School of Materials Science and Engineering Chongqing University of Arts and Sciences Chongqing 402160 P. R. China

**Keywords:** cathode modification, device stability, organic solar cells, photovoltaic efficiency, semiconductor materials

## Abstract

Cathode interlayers (CILs) play a crucial role in improving the photovoltaic efficiency and stability of OSCs. CILs generally consists of two kinds of materials, interfacial dipole‐based CILs and SPS‐based CILs. With good charge transporting ability, excellent compatibility with large‐area processing methods, and highly tunable optoelectronic properties, the SPS‐based CILs exhibit remarkable superiorities to their interfacial dipole‐based counterparts in practical use, making them promising candidate in developing efficient CILs for OSCs. This mini‐review highlights the great potential of SPS‐based CILs in OSC applications and elucidates the working mechanism and material design strategy of SPS materials. Afterward, the SPS‐based CIL materials are summarized and discussed in four sections, including organic small molecules, conjugated polymers, nonconjugated polymers, and TMOs. The structure‐property‐performance relationship of SPS‐based CIL materials is revealed, which may provide readers new insight into the molecular design of SPS‐based CILs. The mechanisms to endow SPS‐based CILs with thickness insensitivity, resistance to environmental erosion, and photo‐electric conversion ability are also elucidated. Finally, after a brief summary, the remaining issues and the prospects of SPS‐based CILs are suggested.

## Graphical abstract

This mini‐review highlights the great potential of solution‐processed semiconductor (SPS) materials as cathode interlayers (CILs) in organic solar cells. The working mechanism and material design strategy of SPS‐based CIL materials are elucidated. The SPS‐based CIL materials, including organic small molecules, conjugated polymers, non‐conjugated polymers, and transition metal oxides, are summarized and the structure‐property‐performance relationship of SPS‐based CIL materials is revealed. After a brief summary, the remaining issues and the prospects of SPS‐based CILs for organic solar cells are suggested.

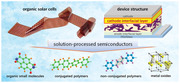

## 1 Introduction

Organic solar cells (OSCs) have attracted extensive attention owing to their light weight, mechanical flexibility, and outstanding potential in large‐area printing.^[^ ^1^, ^2^, ^3^, ^4^, ^5^, ^6^ ^]^ With the rapid advances in the design of high‐performance organic semiconductors, the power conversion efficiencies (PCEs) of single‐junction OSCs have exceeded a landmark value of 19%, showing great promise for commercialization.^[^ ^7^, ^8^, ^9^, ^10^, ^11^, ^12^, ^13^, ^14^ ^]^ A typical OSC device is generally fabricated with a multilayered structure, where a bulk heterojunction (BHJ) active layer containing a bicontinuous interpenetrating network of a donor and an acceptor is sandwiched between two charge‐extracting electrodes.^[^ ^15^, ^16^, ^17^ ^]^ Due to the different electronic structures, the contact interface between organic layer and electrode exhibits large energy barrier for charge collection. Thus, an electrode interlayer has to be utilized to reduce the energy level difference between the electrode and the active layer.^[^ ^18^, ^19^ ^]^ The utilization of electrode interlayers has been proved to be an effective approach to improve the PCE and long‐term stability of OSCs.

Solution‐processed semiconductors (SPSs) have been widely used as electrode interlayers in OSCs due to their advantages of being processed through the low‐cost solution methods.^[^ ^20^, ^21^, ^22^, ^23^, ^24^, ^25^, ^26^ ^]^ The tunable optoelectronic characteristics of SPS materials provide great opportunities to decrease the energy barrier at the electrode/active layer interface, making them more effective in improving OSC performances. More importantly, compared to the electrode interlayers constructed based on the interfacial dipole mechanism, the SPSs possess excellent charge transporting ability, which may effectively suppress the problem of charge accumulation when the interlayer thickness increases.^[^ ^27^, ^28^ ^]^ As a result, the performance of SPS‐based interlayers can exhibit great tolerance to the thickness variation, endowing them with good compatibility with large‐area processing methods, such as coatings and printings, in which certain thickness variation is unavoidable.^[^ ^29^, ^30^, ^31^ ^]^ At present, the conducting polymer PDEDOT:PSS and transition metal oxides (TMOs) MoO_3_ are the most dominantly used anode interlayers, producing many high PCE values for the field.^[^ ^32^, ^33^, ^34^, ^35^, ^36^ ^]^ Meanwhile, a great variety of SPSs are also developed as CILs to facilitate the electron collections in OSCs, including organic small molecules, polymers, and inorganic compounds.^[^ ^37^, ^38^, ^39^ ^]^ To date, many research results have proved that the development of SPS‐based CILs could improve both the PCE and stability of OSCs.^[^ ^40^, ^41^, ^42^ ^]^ However, although numerous SPS materials have been created every year, the underlying mechanism of SPS‐based CILs in improving the OSC performances is still unclear.^[^ ^43^, ^44^ ^]^ In particular, the relationship between molecular structure and modification performance for SPS‐based CILs has not been systematically summarized. Also, the advantages of SPS‐based CILs for practical use need to be further explored and demonstrated so as to provide more insight into how we improve the OSC performances by utilizing advanced CILs. The lack of in‐depth understanding on the properties and advantages of HTLs has become a bigger hurdle in developing efficient CILs. Thus, an overview of the state‐of‐the‐art SPS‐based CILs materials is strongly desired to give the readers an insight into the structure‐property‐performance relationship of CIL materials, proposing promising trends for the development of high‐performance CILs.

**Scheme 1:**
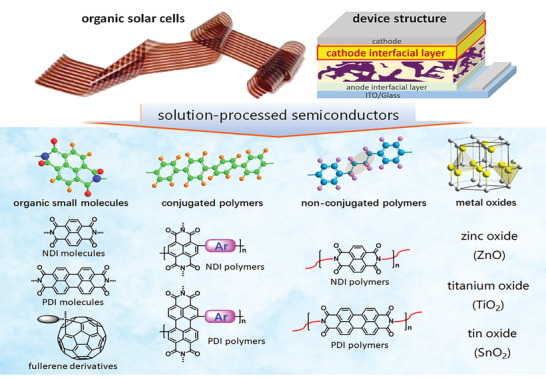
The four catalogues of solution‐processed semiconductors that are most commonly used in OSCs.

In this review, we summarize the most recent progresses of SPS materials as CILs for OSCs. Initially, the working mechanisms of CILs are comprehensively introduced and the superiorities of SPS‐based CILs in practical uses are elucidated and highlighted. Afterward, the SPS‐based CIL materials are discussed in four sections, as shown in **Scheme** **1**, including organic small molecules, conjugated polymers, non‐conjugated polymers, and TMOs. The structure‐property‐performance relationship of SPS‐based CIL materials is revealed, which may provide new guidelines for developing efficient CILs. The related processing methods to improve the photoelectronic and physical properties of SPS‐based CILs are also illustrated, along with their device performances. Moreover, for each kind of SPS‐based CIL, the advantages and the existing problems are discussed. Finally, following a brief summary, we reveal the fundamental challenges and future prospects of SPS‐based CIL materials.

## 2 Working Mechanism of CILs

### 2.1 The Superiority of Semiconductor Materials to Interfacial Dipole‐Based Materials in Serving as CILs for OSCs

**Figure 1:**
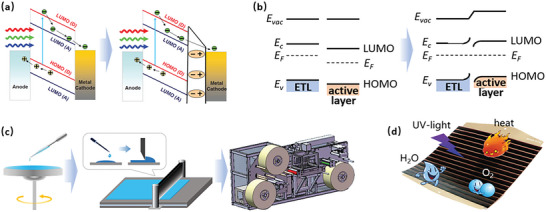
a) Cathode modification by interfacial electrical dipole. Reproduced with permission.^[^ ^45^ ^]^ Copyright 2011, Wiley‐VCH. b) Fermi‐pinning process to facilitate electron transport in the cathode modification. Reproduced with permission.^[^ ^58^ ^]^ Copyright 2009, Wiley‐VCH. c) The commonly used processing methods of spin‐coating, blade‐coating, and roll‐to‐roll printing for preparing CIL. d) The Environmental factors such as ultraviolet light, moisture ingress, oxygen, and heat to deteriorate OSC performance.

Most CILs in OSCs are developed based on two principles. One is the construction of interfacial electrical dipole to facilitate electron extraction, while the other is to develop SPSs with suitable energy level (**Figure** **1**a).^[^ ^45^, ^46^, ^47^, ^48^ ^]^ Many CIL materials are developed based on the interfacial electrical dipole, such as PEIE,^[^ ^49^ ^]^ PVP,^[^ ^50^ ^]^ and MSAPBS.^[^ ^51^, ^52^ ^]^ As one of the most widely used CIL materials, PFN can form a layer of interfacial dipole due to the strong interaction and alignment of polar amino group at the side chain of PFN with the active layer.^[^ ^53^ ^]^ The interfacial dipole not only enhances the built‐in electrical field across the device, but also exerts a strong electrical field at the active layer/cathode interface that may strongly facilitate charge transport and extraction. However, the CILs constructed with interfacial dipole have a fatal shortcoming. The interfacial dipole can only work in ultrathin films (film thickness <5 nm), so the CIL thickness has to be strictly controlled below 5 nm. Moreover, since the dipole‐based CIL enables electron transport through tunneling effect, which only works well within several nanometers, a slight increasing of film thickness will lead to serious charge accumulation and charge recombination. In large‐area processing, the variation of film thickness may reach ±10 nm, which severely degrades the performance of dipole‐based CILs due to the non‐constant film thickness.^[^ ^54^ ^]^ This excludes the application of most dipole‐based CILs in the large‐area fabrication.

Compared to dipole‐based CILs, the SPS‐based CILs have great tolerance to the film thickness variation. Many SPS‐based CILs can work well in the broad thickness range from several nanometers to one micrometer.^[^ ^55^, ^56^, ^57^ ^]^ The excellent thickness insensitivity of SPS‐based CILs is mainly ascribed to two reasons. First, the SPS‐based CILs regulate the interfacial energy level alignment to decrease the barrier for electron extraction through the Fermi‐pinning process, which is independent on film thickness. The Fermi‐pinning process changes the contact at the anode/active layer interface from Schottky contact to ohmic contact, decreasing the energy barrier for electron collection as shown in Figure 1b.^[^ ^58^, ^59^ ^]^ Second, most SPS materials possess good charge transport property, which can effectively suppress the charge accumulation and charge recombination when the CIL thickness increases. Consequently, the performance of SPS‐based CILs is insensitive to film thickness, making them significantly superior to the dipole‐based CILs for practical uses. Due to the good thickness insensitivity, many SPS‐based CILs exhibit excellent compatibility with large‐area processing methods, such as slot‐die coating, blade‐coating, and R2R printing, displaying promising potential for industrial production.^[^ ^60^, ^61^ ^]^

Besides the thickness insensitivity, the structure diversity of SPS materials provides large advantage in tuning the chemical, optical, and electrical properties to improve the electron collection ability of CILs. For instance, the *n*‐doping approach has been proved to enhance the conductivity of SPS‐based CILs, by which the simultaneous improvement of *J*_sc_ and FF can be obtained in OSCs.^[^ ^62^, ^63^ ^]^ Furthermore, the chemical modification by functional groups can modulate the energy level of SPS materials, which reduces the interfacial barrier and increases the *V*_oc_ of the OSC device. Recently, it has also demonstrated that, through rational molecular design, the SPS‐based CILs can act as electron acceptors, contributing to the additional photocurrents for OSCs.^[^ ^64^, ^65^ ^]^ In addition to photo‐electric conversion efficiency, several SPS‐based CILs are endowed with good resistance to moisture ingress and ultraviolet (UV)‐light damage, which greatly improve the long‐term stability of OSCs under operational conditions.^[^ ^66^, ^67^ ^]^ Overall, the diversity in material design and chemical modification endows SPS materials with great opportunities to optimize their performances, making the SPSs promising candidates in developing efficient CIL materials.

### 2.2 The Molecular Design of CILs

An ideal CIL should possess the following characteristic: 1) A low work function (WF, the distance between Fermi level and vacuum level) to decrease the barrier between the cathode and the active layer; 2) a high conductivity to efficiently transport the extracted electrons to the cathode; 3) good transparency to ensure the light‐harvesting of active layer.^[^ ^68^, ^69^ ^]^ In particular, the sufficient ability to decrease the cathode WF is the most essential characteristic for the CIL to selectively extract electrons from the active layer. According to the integer charge‐transfer (ICT) model, when the WF of CIL is lower than the negative integer charge‐transfer state (*E*_ICT_^−^) of acceptor, a spontaneous electron transfer from CIL to acceptor will occur until the Fermi level of CIL is pinned to the negative integer charge‐transfer state at the interface.^[^ ^19^, ^59^, ^70^ ^]^ This helps to form an Ohmic contact at the CIL/active layer interface, so the electrons can transfer across the interface with the minimum of energy loss. This ICT model is particularly suitable to describe the type of weakly interacting interfaces, such as the interfaces in multilayers deposited through solution processing. The ability of CIL to decrease the WF of cathode may be influenced by many factors. First, the molecular orientation arrangement is proved to effectively decrease the WF of cathode, which has been widely used for developing both interfacial dipole‐based and SPS‐based CILs.^[^ ^52^, ^53^ ^]^ Second, the electron‐withdrawing groups are favorable to generate an up‐shift of Fermi level, with respect to the vacuum level, leading to a decrease of WF.^[^ ^71^ ^]^ Particularly, the introduction of electron‐withdrawing groups can not only improve the ability of CIL in decreasing WF, but also modulate the LUMO level of SPS to facilitate electron transport.^[^ ^72^ ^]^ This strategy provides great opportunities to optimize optoelectronic properties of SPS‐based CIL materials through rational molecular design and shows excellent compatibility in improving CIL performances. Third, the *n*‐doping treatment can greatly increase the electron density of SPS materials, leading to an up‐shift of Fermi level (a decline of WF value).^[^ ^73^ ^]^ Fourth, processing methods are also found to influence the WF of CIL by modulating crystal grain size, grain‐size distribution, surface roughness, and attached counterions.^[^ ^74^ ^]^ The mechanism of processing methods in influencing the WF of CIL involve complex factors and has not been systematically studied at the current stage.

Besides the optoelectronic properties, other chemical and physical properties, as well as the preparation cost, should also be considered in the design of CIL materials to improve the photovoltaic efficiency and stability of OSCs. First, the CILs are required to be compatible with large‐area processing methods and can be readily deposited as uniform and smooth films, which depresses the interfacial defects in the multilayer deposition. Generally, polymer CILs possess superior film‐forming ability to their counterparts based on organic small molecules and TMOs. Moreover, as is well known, a high conductivity can effectively suppress the charge accumulation and charge recombination when the CIL thickness is increased, making the CIL performances insensitive to thickness variation.^[^ ^75^, ^76^ ^]^ Thus, the high conductivity should be required in developing CIL of good compatibility with large‐area printing techniques where certain thickness variation is unavoidable. Secondly, it is recognized that the function of improving OSC stability should be considered as an important factor in the design of CIL materials. Under the operational condition, ultraviolet (UV)‐light, moisture, and heat constitute the main detrimental factors to compromise the OSC lifetime. The UV irradiation in sunlight may cause serious photobleaching problem of organic photovoltaic materials, resulting in the deterioration of device performance.^[^ ^77^, ^78^ ^]^ Although the use of a UV filter may help reduce the UV damage, the installation of an additional optical filter not only complicates the structure of the device but also has a negative effect on the light harvesting of OSCs. Thus, CIL materials with negligible photocatalysis activity are desired. Furthermore, the development of CILs that can strongly absorb UV‐light and convert the absorbed UV‐light to an additional photocurrent will be a promising approach to improve the operational stability of OSCs.^[^ ^79^ ^]^ For moisture ingress, it is revealed that CILs with improved hydrophobicity can effectively depress the permeation of water molecule into the interior of device, endowing OSC with great moisture resistance.^[^ ^80^ ^]^ The researches on heat resistance related to CIL are rarely reported, and current results indicate that the thermal instability of OSCs mainly originates from the phase separation of active layers. At present, the utilization of CIL to protect the device against the corrosions from environmental factors has become an important approach to improve the long‐term stability of OSCs under the operational conditions.

## 3 Solution Processable CIL Materials for OSCs

### 3.1 Organic Small Molecules

Organic small molecules show promising potential in serving as CILs with their advantages of easy synthesis, definite structure, and good reproducibility. In particular, the definite chemical structure of organic small molecule can not only ensure the reproducibility of device performance, but also provide an opportunity to investigate the “molecular structure‐photoelectronic property‐device performance” relationship. However, compared to polymers, the film‐forming ability of organic small molecules needs to be further improved so as to depress interfacial defects in OSCs. The CIL materials based on organic small molecules mainly include the derivatives of naphthalene diimide (NDI), perylene diimide (PDI), and fullerene. These compounds possess high electron mobility and low HOMO level, which is essential to be used as CILs.

#### 3.1.1 NDI‐Based Molecules

NDI‐based molecules have been developed as efficient CIL materials due to its adjustable energy level, high electrical conductivity, and easy modification of chemical structure.^[^ ^81^, ^82^, ^83^, ^84^ ^]^ Compared to their PDI counterparts, NDI‐based CILs possess much higher optical transmittance, which is favorable to the light‐harvesting of active layer. Through rational molecule design, the chemical stability, molecular packing, and processing property of NDI molecules can be effectively modulated, providing diverse approaches to improve the performance of CILs.

**Figure 2:**
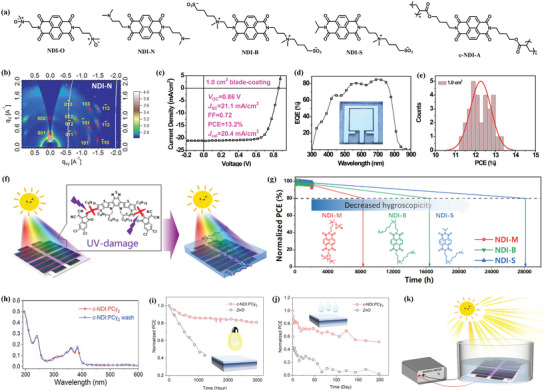
a) Chemical structures of NDI‐based CIL molecules. b) 2D GIWAXS patterns for NDI‐N. c) *J–V* and d) EQE curves of devices using blade‐coated NDI‐N as CIL. e) Histograms of the PCE counts for 30 individual 1.0‐cm^2^ cells. Reproduced with permission.^[^ ^86^ ^]^ Copyright 2019, Elsevier. f) Diagram of UV‐visible absorption of NDI‐B.^[^ ^79^ ^]^ Copyright 2021, Chinese Chemical Society. g) PCE of NDI‐M, NDI‐B, and NDI‐S over time. h)The UV‐vis absorption spectra c‐NDI:PCy_2_ before (red line) and after (blue line) chloroform washing. i) Normalized PCE values as a function of time of being soaked in water under illumination for non‐encapsulated ZnO, NDI‐B, PEDOT:PSS, and c‐NDIPCy_2_‐modified devices. j) Normalized PCE values as a function of storage time of being soaked in water under dark for ZnO and c‐NDI‐PCy_2_‐based devices without encapsulation. k) The schematic diagram of the in situ test of OSC in water. Reproduced with permission.^[^ ^88^ ^]^ Copyright 2023, Elsevier.

In 2015, Hou and co‐workers synthesized a CIL material NDIO based on the NDI unit.^[^ ^85^ ^]^ The NDIO exhibits high transparency, good water solubility, and suitable work function for efficient electron collection. By using the PBDT‐TS1:PC_71_BM blend as active layer, the OSC modified by NDIO shows a PCE of 9.51%. Besides, the PCE of PBDTTT‐EFT:N2200‐based OSC can be increased from 3.23% to 5.77% by incorporating NDIO. After that, the same group reports another NDI‐based small molecule NDI‐N that has both high crystallinity and excellent film forming ability are exhibited in **Figure** **2**b, endowing the CIL with excellent electron transport properties and good processability.^[^ ^86^ ^]^ The chemical structure of NDI‐N is presented in Figure 2a. The NDI‐N exhibits unique superiority to common CIL materials due to its good compatibility with large‐area processing methods. By using a blade‐coated NDI‐N as CIL, a large area OSCs device with an area of 1 cm^2^ was fabricated (Figure 2e), showing a PCE of 13.2% (Figure 2c). The results indicate that the development of printable CILs paves new way for low‐cost and mass production of OSCs.

Besides photovoltaic efficiency, long‐term stability is another key factor for the practical use of organic photovoltaic technology. The development of CILs plays an important role in improving the stability of OSCs. Many results indicate that the ultraviolet (UV) ray from sunlight may cause decomposition of organic photovoltaic compounds, making OSCs intrinsically unstable under sunlight illumination. To solve this problem, Xu et al.^[^ ^79^ ^]^ designed and synthesized a UV‐resistant CIL, namely NDI‐B, to improve the operational stability of OSCs. As shown in Figure 2f, the NDI‐B zwitterion can convert the absorbed high‐energy UV photons to charge carriers, which not only increases the photocurrent of the device but also protects the photoactive layer from UV‐induced decomposition. The OSC with NDI‐B exhibits a PCE of 17.2%, and the device exhibits a T80 (a lifetime parameter defined as the time over which the PCE decays to 80% of its initial value) of over 1800 h under full‐sun AM 1.5 G illumination. Furthermore, through rational molecular design, Liao and co‐workers synthesize NDI‐S with low hygroscopicity to prevent moisture ingress, by which the OSC stability under high humidity can be greatly improved.^[^ ^87^ ^]^ The OSC modified by NDI‐S exhibited a PCE of 17.3%, along with a T80 lifetime of over 28 000 h (Figure 2g). Recently, Yang et al.^[^ ^88^ ^]^ design a cross‐linkable NDI derivative to prepare a robust (Figure 2h) and hydrophobic film *c*‐NDI:PCy_2_ as CIL for OSCs. Through a simple two‐step route, an acrylate‐modified organic small molecule NDI‐A is synthesized as the precursor, and can be easily prepared as a cross‐linked c‐NDI:PCy2 film by the thermal‐annealing treatment. By using a non‐polar electron donor dicyclohexyl(2′,6′‐dimethoxy‐[1,1′‐biphenyl]−2‐yl)‐phosphine (PCy_2_) as dopant, a high conductivity of 6.51 × 10^−3^ S m^−1^ can be achieved in the *c*‐NDI:PCy_2_ film, which is essential for electron collection. The strong hydrophobicity of *c*‐NDI:PCy_2_ effectively protects the device against water ingress, endowing OSCs with excellent water‐resistance. Based on the *c*‐NDI:PCy_2_ CIL, a PCE of 17.7% can be obtained in the OSCs. Intriguingly, the *c*‐NDI:PCy_2_‐modified OSC can be used underwater. By immersing the non‐encapsulated cell in water, 70% of its initial efficiency is maintained after 1000 hours in the dark (Figure 2j) or 4 hours under continuous illumination (100 mW cm^−2^) (Figure 2i).

#### 3.1.2 PDI‐Based Molecules

**Figure 3:**
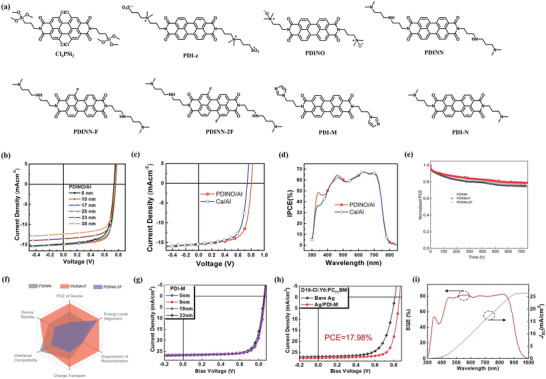
a) Chemical structures of PDI‐based CIL molecules. b) PDINO interlayers with various thicknesses. c) *J*–*V* curves of the PSCs based on PTB7‐Th/PC_70_BM with Ca/Al or PDINO/Al as cathodes together with d) the corresponding IPCE spectra. Reproduced with permission.^[^ ^91^ ^]^ Copyright 2014, Royal Society of Chemistry. e) Normalized PCE plotted against ageing time under 1‐Sun illumination from white LED for representative devices. f) Radar chart for the comparison of the performance of three PDIs in various physical measurements. Reproduced with permission.^[^ ^93^ ^]^ Copyright 2022, Wiley‐VCH. g) Devices containing the PDI‐M interlayer of different thicknesses with the PM6:Y6 active layer. h) D18‐Cl: Y6:PC_71_BM‐based OSCs containing no interlayer (bare Ag) or PDI‐M with optimized thickness. i) EQE spectrum of the D18‐Cl: Y6:PC_71_BM ternary OSCs containing PDI‐M. Reproduced with permission.^[^ ^94^ ^]^ Copyright 2021, American Chemical Society.

Although PDI‐based molecules exhibit higher absorption in visible region, compared to the NDI compounds, due to their large planar π‐conjugated structure, the reinforced *π*–*π* interaction promote the intermolecular stacking, endowing the PDI molecules with high electron transport ability. Benefitting from the excellent electron transporting property, the PDI derivatives exhibit great advantage in developing printable CILs that possess good compatibility with large‐area roll‐to‐roll processing where certain thickness variation is unavoidable. It should be noted that the large planar frame of PDI unit may lead to high crystallinity of its derivatives, which is unfavorable to form smooth CIL film. The molecular structures of several representative PDI‐based CILs are shown in **Figure** **3**a. Through rational side‐chain engineering, the crystallinity of PFDI derivatives can be modulated to improve the film‐forming ability.

In 2011, Hains and co‐workers reported a PDI‐based small‐molecule CIL Cl_4_PSi_2_ that can be covalently attached on ITO substrate by the two successive processes of spin‐coating and thermal‐induced cross‐linking.^[^ ^89^ ^]^ The LUMO level of Cl_4_PSi_2_ (−4.5 eV) matches well with the active layer material, which is beneficial to electron extraction and transport. With the P3HT:PCBM active layer, the OSCs modified by the cross‐linked show a PCE of 1.8%. Moreover, Fang et al.^[^ ^90^ ^]^ reports a water/alcohol‐soluble perylene diimide zwitterion (PDI‐z) consisting of sulfobetaine ion as a terminal substituent and PDI as a conjugated core. The PDI‐z‐modified OSCs displayed excellent photovoltaic performances over a wide range (5–40 nm) of CIL thicknesses, and a PCE of 11.23% was obtained with a high FF of 74.2% for the PBDB‐T:IT‐M device.

As a classic CIL material, PDNIO was designed and synthesized by Zhang and co‐workers by using amino N‐oxides end‐substituents to modify the PDI unit.^[^ ^91^ ^]^ The CIL PDINO exhibit a low work function of 3.6 eV and a high electrical conductivity of 7.6 × 10^−5^ S cm^−1^. The high conductivity of PDINO effectively solves the problem of charge accumulation and charge recombination when the CIL thickness increases, endowing the CIL with excellent tolerance to thickness variation. When the PDINO thickness is increased from 6 nm to 30 nm, as depicted in Figure 3b, only a slight decline of PCE from 8.24% to 7.22% can be observed in OSCs, indicating the good thickness insensitivity of PDINO (Figure 3c,d). Although PDNIO displays excellent performance in serving as CIL in OSCs, the strong polar amino N‐oxides group may result in a high surface energy and poor physical contact at the active layer surface, posing a challenge for interlayer engineering to address the trade‐off between PCE and device stability. Thus, Zhang et al.^[^ ^92^ ^]^ designed and synthesized a hydrogen‐bonding interfacial material PDINN that can simultaneously down‐shift the work function of the cathodes (silver and copper) and maintain good interfacial contact with the active layer. The hydrogen‐bonding feature of PDINN can improve the interfacial compatibility, with the active layer below through a spontaneous adsorption process driven by the hydrogen‐bonding interactions, thus reduce the series resistance of OSCs. The OSC modified by PDINN shows a high PCE of 17.23% (certified value 16.77% by NREL) and high stability. To further optimize the energy level structure of PDINN, Zhang and co‐workers designed and synthesized two fluorinated PDI derivatives PDINN‐F and PDINN‐2F through a simple fluorination method, for the application as CIL materials in OSCs.^[^ ^93^ ^]^ The bay‐fluorinated PDI compounds possess low LUMO level of ≈−4.0 eV, which matches well with the high‐performance organic electron acceptor such as BTP‐eC9 for a favorable electron extraction efficiency. The PDINN‐F‐modified OSC with the PM6:BTP‐eC9 active layer exhibited a high PCE of 18.02%, along with an FF of 0.78. In addition, the operational stability of the OSC can also be improved by using the PDINN‐F CIL, retaining more than 80% of its initial PCE after operating at the maximum power point under continuous illumination for 750 h (Figure 3e).

Many research results suggest that the planar backbones of PDI derivatives may lead to coarse CIL surfaces due to the high crystallinity, resulting in the formation of interfacial defects. To solve this problem, Liu and co‐workers synthesized an imidazole‐functionalized PDI molecule PDI‐M, exhibiting high electron affinity and conductivity to effectively reduce the work function of cathode.^[^ ^94^ ^]^ Compared to the amine‐functionalized PDI molecule, the crystallinity of imidazole‐functionalized molecule was moderately suppressed, affording good film‐forming properties. The PDI‐M CIL is not only compatible with various active layers in solar cells, but also show good cathode modification performance over a wide thickness range from ≈5 to ≈33 nm (Figure 3g). The PCE of PDI‐M‐modified OSC can reach 17.98% (Figure 3h,i), which is among the highest PCE values in OSCs. To further improve the electron collection efficiency, Wu et al.^[^ ^95^ ^]^ report the utilizing of acid‐assisted noncovalent interaction between CIL and organic electron acceptor to form the effective electron transport channels. The acetic acid‐treated PDIN provides a PCE of 16.43% in OSCs, showing the enhancement of ≈51% and ≈7% in PCE as compared with the devices modified by the pristine PDIN and PDINO. Similarly, Kan and co‐workers also improve the interaction between the organic electron acceptor and the CIL by developing a hybrid CIL composing of PNDIT‐F3N and PDIN mixed PDIN with PNDIT‐F3N to produce a hybrid CIL.^[^ ^96^ ^]^ The OSC with the hybrid interlayer showed enhanced exciton dissociation, and reduced trap‐assistant recombination compared with those of the devices with PNDIT‐F3N and PDIN. Compared to devices with PNDIT‐F3N and PDIN as the CIL, the device with the hybrid interlayer yielded higher FF of 0.74 and a high PCE of 17.4%.(**Table** **1**).

**Table 1:** Devices parameters of OSCs with Organic small molecules.

CILs	Photoactive layer	*V*_oc_ [V]	*J*_sc_ [mA cm^−2^]	FF	PCE [%]	Ref.
NDIO	PBDT‐TS1:PC_71_BM	0.79	18.02	0.66	9.51	[85]
NDI‐N	PBDB‐T‐2F:IT‐4F	0.86	21.30	0.76	13.90	[86]
NDI‐B	PBDB‐TF :BTP‐eC9	0.84	26.68	0.77	17.23	[79]
NDI‐S	PBDBTF:BTP‐eC9	0.84	26.61	0.77	17.30	[87]
c‐NDI:PCy_2_	PBDB‐TF:BTP‐eC9	0.83	28.30	0.75	17.70	[88]
Cl_4_PSi_2_	P3HT:PCBM	0.48	8.82	0.42	1.80	[89]
PDI‐z	PBDB‐T:IT‐M	0.94	16.12	0.74	11.23	[90]
PDINO	PTB7:PC70BM	0.75	14.29	0.71	11.23	[91]
PDINN	PM6:Y6	0.85	25.89	0.78	17.23	[92]
PDINN‐F	PM6: BTP‐eC9	0.84	27.39	0.78	18.02	[93]
PDINN‐2F	PM6: BTP‐eC9	0.84	26.43	0.76	16.81	[93]
PDI‐M	D18‐Cl: Y6: PC_71_BM	0.86	27.28	0.75	17.98	[94]
PDIN	PBDB‐T‐2F:BTP‐4F	0.86	26.51	0.72	16.43	[95]
PNDIT‐F3N/ PDIN	PM6:Y6	0.86	27.12	0.74	17.40	[96]
B‐PCPO	PCDTBT:PC_71_BM	0.89	9.50	0.61	6.20	[100]
C_60_‐ionene	PBDB‐T:ITIC	0.92	16.57	0.68	11.04	[102]
PCBDAN	P3HT:PCBM	0.58	9.90	0.67	3.85	[103]
DMAPA‐C_60_	N(Ph‐2T‐DCN‐Et) 3 :PC_70_BM	0.96	10.50	0.53	5.40	[104]
FPNOH	PTB7:PC_71_BM	0.75	16.44	0.68	8.34	[105]
PCBSD	P3HT:PCBM	0.60	12.80	0.58	4.40	[106]
Phen‐NaDPO	PTB7:PC_61_BM	0.75	16.81	0.68	8.56	[107]
S‐3	PBDB‐TF:BO‐4Cl	0.84	25.97	0.76	16.56	[109]
OSiNDs	PM6:Y6:PC71BM	0.85	26.02	0.77	17.15	[110]
CTOC‐N‐Br	PM6:BTP‐4Cl	0.84	26.57	0.77	17.19	[111]
SiNcTI‐N	PM6:Y6/CIM/Ag	0.85	25.98	0.75	16.71	[112]
t‐PyDIN	PM6: BTP‐eC9	0.84	28.24	0.76	18.25	[114]
t‐PyDINO	PM6: BTP‐eC9	0.84	27.70	0.75	17.56	[114]
t‐PyDINBr	PM6: BTP‐eC9	0.83	27.84	0.74	17.24	[114]

#### 3.1.3 Fullerene Derivatives (FDs)

**Figure 4:**
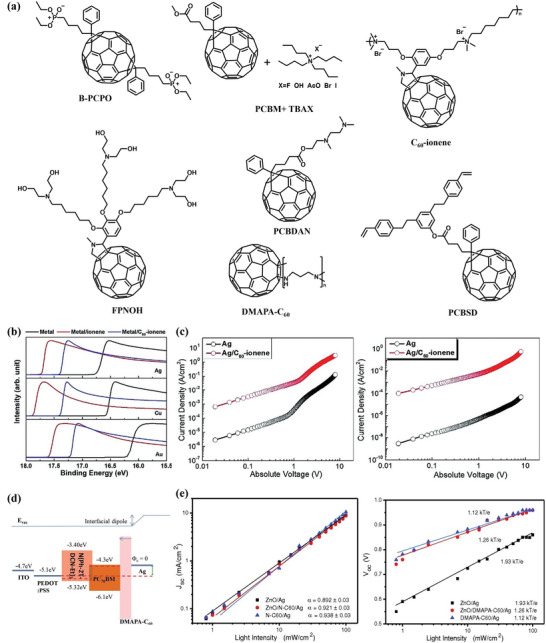
a) Chemical structures of FD‐based CIL molecules. b) UPS spectra comparing bare metals to polymer‐coated metals (Ag, Cu, and Au). c) *J–V* curves of the electron‐only devices employing PC_71_BM and ITIC. Reproduced with permission.^[^ ^102^ ^]^ Copyright 2019, Wiley‐VCH. d) Schematic representation of the band energy diagrams for the conventional configuration of SMOSCs based on DMAPA‐C_60_ /Ag cathode. e) Measured *J_sc_* and *V*_oc_ of devices with ZnO/Ag, ZnO/DMAPA‐C_60_ /Ag, and DMAPA‐C_60_ /Ag cathodes as a function of light intensity, together with linear fits to the data (solid lines). Reproduced with permission.^[^ ^104^ ^]^ Copyright 2014, Wiley‐VCH.

FDs are superior semiconductor materials displaying with excellent properties such as diverse structures, high chemical stability, tunable energy levels, and high electron mobility.^[^ ^97^, ^98^ ^]^ Through diverse chemical modification methods, FDs can have good solubility, which endows the CILs with excellent solution processability. Moreover, with rational molecular design, FD‐based CILs may exhibit suitable energy levels that match well with the LUMO level of electron acceptor so as to facilitate electron extraction and transport.^[^ ^99^ ^]^ Benefitting from the superior charge transport ability, FD‐based CILs can display stable performance for electron collection over a wide thickness range, making them promising candidates in the large‐area fabrication of OSCs. **Figure** **4**a summarizes several typical FD‐based CIL molecules in OSCs.

In 2012, Huang et al.^[^ ^100^ ^]^ reported s phosphate group‐containing bisadducts FD B‐PCPO that can be used to modify ITO electrode in the fabrication of inverted OSCs. The B‐PCPO interlayer can effectively decrease the work function of ITO, improving the electron collection efficiency. As a result, the PCE of OSC can be enhanced from 4.83% to 6.20% by using a B‐PCPO interlayer. As is well known, doping can simultaneously minimize transport loss and shift the Femi level of the semiconductor films, which facilitates Ohmic contact at the CIL/electrode interfaces. Thus, Jen et al.^[^ ^101^ ^]^ demonstrate effective doping of fullerenes via a simple solution‐processed blend of PCBM and ordinary tetrabutylammonium salt (TBAX, X^−^ = F^−^, OH^−^, AcO^−^, Br^−^, I^−^) and achieve a high conductivity of 0.56 S/m in the cast film. The electron transfer (ET) process between the anions of TBAXs and PCBM induces effective *n*‐doping under very mild conditions, which can be further regulated by adjusting the solvent polarity and the state of blend in solution or in solid. The doped PCBM leads to an optimized interfacial contact and results in improvement of OSC efficiency.

To endow FDs with good water/alcohol solubility, Russell and co‐workers integrating C_60_ fullerene monomers into ionene polymers and synthesized C_60_‐ionene as CIL for OSCs.^[^ ^102^ ^]^ As shown in Figure 4b, The C_60_‐ionene displays outstanding ability to decrease the WF of numerous metals (e.g., Ag, Cu, and Au), improving the electron collection property of the cathodes. The researchers proved that the introduction of fullerene moieties could effectively improve the conductivity of ionene polymers (Figure 4c), affording the OSC with high photovoltaic efficiency by reducing the charge recombination at the CIL/cathode interface. Based on the C_60_‐ionene CIL, OSC with a non‐fullerene acceptor in the active layer exhibited a PCE of 11.04%. The C_60_‐ionene also exhibits good performance in cathode modification in a wide thickness range of 2–60 nm.

Chen and co‐workers proposed a new strategy of self‐organization to simultaneously prepare CIL and active layer in one step for fabricating inverted OSCs.^[^ ^103^ ^]^ An amine‐modified alcohol‐soluble FD PCBDAN is designed and synthesized, and can be mixed as an additive in the solution of active layer materials. The similar Lewis base interaction and close surface energies for ITO and PCBDAN could drive PCBDAN toward the buried ITO interface, which spontaneously produce the separation of CIL and active layer. Based on the P3HT:PCBM active layer, an OSC that is fabricated by the one‐step method showed a PCE of 3.85%, indicating the potential of self‐organization method in the OSC fabrication. Min et al.^[^ ^104^ ^]^ further investigated the influence of interface engineering on OSC performance by regulating the interfacial contact among different layers. By inserting an amine group functionalized fullerene complex DMAPA‐C_60_ as a buffer layer to modify the contact properties of ZnO/Ag interface, a better Ohmic contact at the cathode can be formed (Figure 4d), resulting in higher photovoltaic performance of OSCs. The incorporation of the DMAPA‐C_60_ buffer layer effectively reduces the density of traps between the acceptor in the active layer and Ag contact, and hence defect or trap‐assisted interface recombination is suppressed, as shown in Figure 4e, leading to an enhancement of PCE from 2.75% to 4.31% in OSCs. The researchers also constructed a DMAPA‐C_60_/Ag cathode, by which a further PCE increase to 5.40% was obtained, along with an improved device stability.

Aiming at the fabrication of OSCs with inverted structure, Lai et al.^[^ ^105^ ^]^ introduced the diethanolamino group into FPNOH, by which the FD‐based CIL material can be readily deposited on the hydrophilic surface of ITO electrode to form smooth film. The FPNOH exhibits good resistance to the lipophilic solvents, so the CIL can be used for fabricating inverted OSCs. It is also demonstrated that the introduced diethanolamino polar groups can improve the electron extraction ability of the CIL. The FPNOH shows thickness‐insensitive performance in serving as CIL, i.e., when the FPNOH thickness is increased from 6.0 to 16.9 nm, the PCE of OSCs is only slightly decreased from 8.34% to 7.73%. Cross‐linking provides another method to prepare CIL with excellent solvent resistance. As a classic example, Hsu and co‐workers designed and synthesized a PCBM‐based n‐type organic semiconductor material PCBSD containing two styryl groups as thermal cross‐linkers.^[^ ^106^ ^]^ In situ cross‐linking of PCBSD can be realized by heating the precursor PCBSD film at a low temperature of 160 °C for 30 min to generate a robust, adhesive, and solvent‐resistant thin film. With the incorporation of the cross‐linked PCBSD film, the OSC not only exhibits enhanced PCE from 3.5% to 4.4%, but also shows an excellent device lifetime without encapsulation.

#### 3.1.4 Others

Besides the representative organic semiconductors based on NDI, PDI, and fullerene units, other semiconductors with novel structures are also developed as CIL materials in OSCs. These materials not only enhance the photovoltaic performances of OSCs, but also provide more insight into the “chemical structure‐property‐performance” relationship for developing efficient CIL materials.

Tan et al.^[^ ^107^ ^]^ designed and synthesized Phen‐NaDPO by combining triarylphosphine oxide with a 1,10‐phenanthrolinyl unit for the use as CIL in OSCs. The organic semiconductor possesses easy synthesis and purification, high chemical stability and excellent electron transport properties. The coupling of the P = O functional group with Ag leads to the strong interaction between the CIL and the cathode, which significantly reduce the work functions of cathode and promote the electron extraction in OSCs. The OSC device modified by Phen‐NaDPO showed a PCE of 8.56%, which is much higher than that of the reference device (PCE = 7.31%). By blending Phen‐NaDPO into the polymer/fullerene active layer solution and blade‐coating the mixture on ITO/ZnO substrate, Zhu et al.^[^ ^108^ ^]^ observed a spontaneous, surface energy‐driven migration of Phen‐NaDPO towards the ZnO interface and a subsequent formation of ZnO/Phen‐NaDPO bilayer. The self‐organized Phen‐NaDPO layer improves the electron selective and barrier‐free extraction contacts between cathode and active layer, affording a PCE enhancement from 4.0% to 5.2%. This self‐organization approach not only works well with various cathodes such as bare ITO, ITO/ZnO, and ITO/AZO, but also shows good compatibility with large‐area roll‐to‐roll processing method.

**Figure 5:**
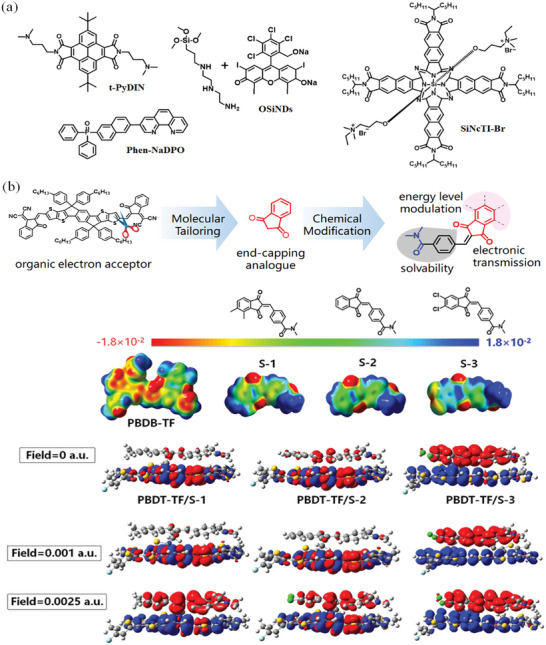
a) Chemical structures of organic small molecules as CILs; b) Organic small molecules as CILs that are developed by the “tailoring electron acceptor” strategy. Reproduced with permission.^[^ ^109^ ^]^ Copyright 2019, Wiley‐VCH.

Xu et al.^[^ ^109^ ^]^ utilize the excellent electron transport property of electron acceptor ITIC and propose a new design strategy of tailoring the end‐capping unit of electron acceptor to develop the CIL material for efficient OSCs (**Figure** **5**b). The synthesized CIL molecules exhibit tunable energy levels from −3.47 to −3.73 eV and good film‐forming ability, endowing the CILs with outstanding electron extraction property. The OSC with S‐3 shows a high PCE of 16.6%. By the combination of density functional theory calculation and experimental results, the researchers also demonstrated that S‐3 can not only efficiently extract electrons from the cathode but also serve as an electron acceptor to promote exciton dissociation at the CIL/active layer interface, contributing to additional photo‐induced current generation. Li et al.^[^ ^110^ ^]^ synthesized a cost‐effective organosilica nanodot OSiNDs via a simple one‐step hydrothermal reaction. The OSiNDs CIL is highly stable under thermal stress or photoillumination (UV and AM 1.5G) and undergoes no photochemical reaction with the photoactive materials. With the incorporation of OSiNDs, the OSC exhibited a high PCE of 17.15% and maintained 96.1% of the initial efficiency after continuous illumination (AM 1.5G, 100 mW cm^−2^) for 600 min. In contrast, the reference device with ZnO remained only 67.2% of its performance under the same condition. Furthermore, Li and co‐workers reported an organic‐inorganic hybrid electrolyte CTOC‐N‐Br containing CTOC as core and naphthalene‐based organic ammonium bromide salts as the electrolyte.^[^ ^111^ ^]^ The hybrid electrolyte exhibits excellent solubility in methanol, high electron mobility (2.80 × 10^−4^ cm^2^ V^−1^ s^−1^), and amorphous state in thin film, film, enabling its application as a CIL in OSCs with a high PCE of 17.19%. Zhang et al.^[^ ^112^ ^]^ synthesized a naphthalocyanine derivative SiNcTI‐Br with the chemical modification by two hydrophilic quaternary ammonium and four electron‐withdrawing imide groups. The SiNcTI‐Br showed deep LUMO energy level below −3.90 eV and strong self‐doping property. The SiNcTI‐Br semiconductor displayed high conductivity of 4.5 × 10^−5^ S cm^−1^ and electron mobility of 7.81 × 10^−5^ cm^2^ V^−1^ s^−1^, which could boost the photovoltaic efficiencies of OSCs over a wide CIL thickness range of 4–25 nm, with the maximum PCE of 16.71%.

As is well‐known, the *n*‐doping has been recognized as an essential factor to enable Ohmic‐like contact between the electrode and organic layers and thus promote the performances of organic optoelectronic devices. Thus, Yang et al.^[^ ^113^ ^]^ synthesized multifarious azaphenalene‐embedded organic salts to develop new CIL materials with improved n‐type self‐doping property, via introducing variable counterions, substituent groups, and repeating units. Through a counterion‐induced proton transfer process, diradicaloids were formed and delocalized over the *π*‐conjugated systems, which is beneficial to enhance the carrier density of the matrix and remarkably decrease the work functions of the Al electrode. By using these azaphenalene‐based molecules as CILs, the all‐solution‐processed OSC can be fabricated, which results in high PCE of 10.24% in contrast to the 7.34% of the reference device without CILs. To explore the use of organic semiconductors as CILs, Zhang's group constructs a highly efficient CIL based on the 2,7‐di‐*tert*‐butyl‐4,5,9,10‐pyrene diimide (t‐PyDI) framework.^[^ ^114^ ^]^ Three small molecules t‐PyDIN, t‐PyDINO, and t‐PyDINBr with self‐doping property were synthesized by modifying the t‐PyDI unit with amino, amino n‐oxide, and quaternary ammonium bromide, respectively. Among the three compounds, t‐PyDIN exhibits high thermal stability, and helps the OSC to achieve a high PCE of 18.25%, representing the state‐of‐the‐art device in the field. The result suggests that, due to the diverse structures and plentiful chemical modification methods, organic semiconductors have promising potential to be developed as efficient CIL materials for OSCs.

### 3.2 Conjugated Polymers (CPs)

**Figure 6:**
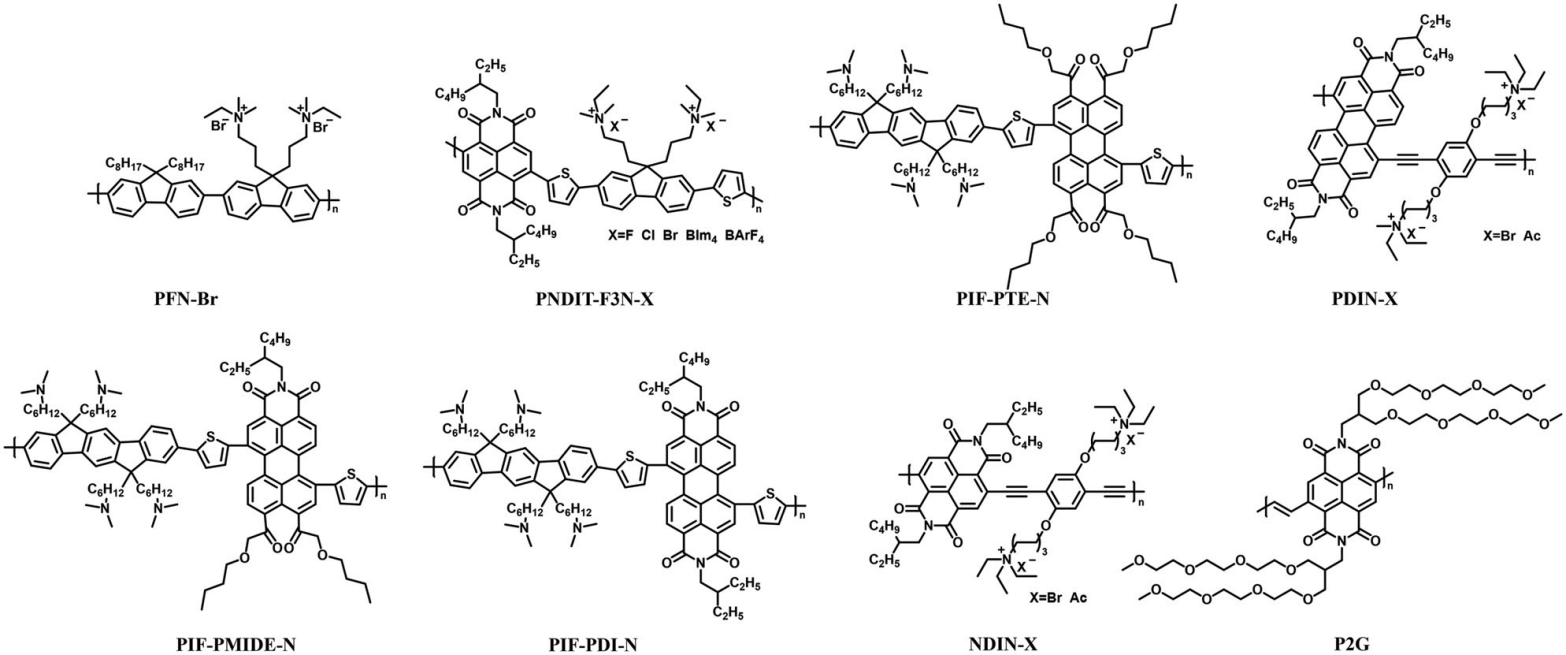
Chemical structures of conjugated polymers as CILs.

Conjugated polymers have excellent electron‐transport property and good film‐forming ability, showing great advantage in cathode modification.^[^ ^115^, ^116^ ^]^ In particular, the good charge carrier transport property greatly suppresses the problem of charge recombination as the CP thickness is increased. As a result, CPs usually exhibit thickness‐insensitive performance in serving as CIL, making them compatible with large‐area processing methods, such as blade‐coating, slot‐die‐coating, and roll‐to‐roll printing.^[^ ^117^, ^118^, ^119^ ^]^ This is essentially important for commercialization and large‐scale manufacturing of OSCs. In addition, the chemical structure of CPs can be readily modified through organic synthesis, providing great opportunities to improve CIL performances by tuning the chemical and optoelectronic properties. At present, most CP‐based CILs are developed based on PDI and NDI units, and their structures are presented in **Figure** **6**. In recent years, the exploration of solution processable CPs for efficient CIL materials has become a more and more important approach for promoting OSC performances.

**Figure 7:**
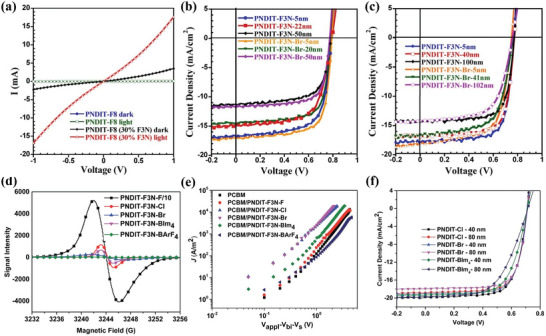
a) *J*–*V* curves of devices for the blend film PNDIT‐F8/F3N in dark and under illumination. b) *J*–*V* curves of PSC devices using PTB7‐Th:PC_71_BM as the active layer with different CILs in various thickness. c) *J*–*V* curves of PSC devices using PffBT4T‐2OD:PC_71_BM as the active layer with different CILs in various thickness. Reproduced with permission.^[^ ^120^ ^]^ Copyright 2016, American Chemical Society. d) The ESR curves of PNDIT‐F3N‐Xs in the solid state. e) *J*–*V* curves of electron‐only devices with the structure of ITO /Al / PC_71_BM / CPEs (5 nm)/Al. f) *J*–*V* curves of photovoltaic devices with 40 nm or 80 nm CPEs. Reproduced with permission.^[^ ^122^ ^]^ Copyright 2017, Royal Society of Chemistry.

With great superiority in electron extraction, PFN‐Br has been dominantly used as CIL material in OSCs for more than ten years. However, PFN‐Br can only work well with an extremely thin layer of ≈5 nm, which is difficult to realize in industrial production. To solve this problem, Huang et al.^[^ ^120^ ^]^ designed and synthesized two *n*‐type water/alcohol‐soluble CPs PNDIT‐F3N and PNDIT‐F3N‐Br based on the NDI unit. The photo‐induced intramolecular electron transfer from amino groups to the conjugated backbone of the polymer leads to a significant enhancement of conductivity for PNDIT‐F3N (**Figure** **7**a). As a result, OSC based on the PffBT4T‐2OD/PC_71_BM active layer could exhibit a PCE of 10.11%. The researchers also proved that, since the LUMO of NDI‐based CPs matches well with most organic/polymer electron acceptors, charge can be injected into the CPs and subsequently transport within the CIL films, so there is no electron barrier formed between the CIL/active layer interface. Consequently, a large thickness window of CPs PNDIT‐F3N and PNDIT‐F3N‐Br can be applied to achieve high photovoltaic performances in OSCs without affecting the electron extraction properties at the cathode interface. As shown in Figure 7b,c, it is worth noting that OSCs modified by PNDIT‐F3N and PNDIT‐F3N‐Br with film thicknesses of 100 nm can still show PCE of 7.97% and 8.04%, respectively. Recently, Huang et al.^[^ ^121^ ^]^ changed the electron‐deficient blocks in the backbones of CPs based on different perylenetetracarboxylic acid units and synthesized three CPs PIF‐PTE‐N, PIF‐PMIDE‐N, and PIF‐PDI‐N for the applications as CIL materials. The distinct nature of the electron‐deficient blocks in polymer backbones endows the three CPs with tunable absorptions and energy levels. The strong *n*‐doping effect and favorable main‐chain stacking result in the high electron mobility of PIF‐PDI‐N, which helps to realize a PCE of 10% in OSCs. When the PIF‐PDI‐N thickness is increased to 50 nm, a PCE over 9% is still remained for OSC with the PNTT/PC_71_BM active layer, implying a possibility to fabricate large‐area OSCs via the R2R method.

To further improve the performance of CILs, Huang et al.^[^ ^122^ ^]^ investigate the influence of size, type, and substituent of counterions on optoelectronic properties of CPs. Different counterions (F^−^, Cl^−^, BIm4^−^ and BArF4^−^) were introduced in PNDIT‐F3N‐Br derivatives as side chains through ion exchange. The results suggest that counterions with strong electron‐withdrawing substituent groups can preclude the self‐doping behaviors of CPs, increase the work function of cathodes, and impede electron transport (Figure 7e). With optimized counterions, CPs can possess higher electron mobility, exhibiting more potential as thickness‐insensitive CILs for the large‐area fabrication of OSCs (Figure 7f). Besides, different nonionic polar groups such as dimethylamino, *N*‐methylbenzylamino, and dibenzylamino were also introduced as pendants into NDI‐based CPs.^[^ ^123^ ^]^ The conjugation effect and steric hindrance correlated significantly with the electron‐donating ability of the polar side chain, which has essential influence on the self‐doping property of the NDI‐based CPs. By introducing the rigid acetylene spacer to the polymer skeleton, the electron transport property of the CP can be effectively improved, realizing a PCE enhancement from 9.45% (without acetylene structure) to 10.09% (with acetylene structure). Based on the PNDIT‐F3N‐Br category, Huang's group systematically studied the relationship between chemical structure and doping property in CPs by synthesizing a series of CPs NDIN‐Br, NDIN‐Ac, PDIN‐Br, and PDIN‐Ac with tailored backbones and polar groups. ^[^ ^124^ ^]^ The results revealed that the doping effect and photoconductivity of *n*‐type CPs are significantly influenced by the electron affinities of conjugated backbones and electron‐donating strengths of the pendant polar groups. The OSC with NDIN‐Br and PDIN‐Br shows the best PCEs of 9.88% and 9.83%, respectively. Baran group synthesized P2G based on the NDI core and ethylene glycol side chain.^[^ ^125^ ^]^ The P2G not only possesses good alcohol solubility and high thermal stability, but also exhibits appropriate molecular energy level for electron collection and high conductivity of 2.3 × 10^−6^ S cm^−1^. The OSC based on the PM6:Y6 active layer shows a PCE of 16%. In addition, P2G can be deposited via printing methods, showing promising prospects in the roll‐to‐roll fabrication of OSCs.(**Table** **2**).

**Table 2:** Devices parameters of OSCs with CPs and NCPs.

CILs	Photoactive layer	*V*_oc_ [V]	*J*_sc_ [mA cm^−2^]	FF	PCE [%]	Ref.
PNDIT‐F3N	PffBT4T‐2OD:PC_71_BM	0.75	18.38	0.65	9.42	[120]
PNDIT‐F3N‐Br	PffBT4T‐2OD:PC_71_BM	0.77	17.64	0.72	10.11	[120]
PIF‐PTE‐N	PNTT:PC_71_BM	0.75	19.44	0.69	10.31	[121]
PIF‐PMIDE‐N	PNTT:PC_71_BM	0.75	19.48	0.68	10.21	[121]
PIF‐PDI‐N	PNTT:PC_71_BM	0.75	19.73	0.68	10.49	[121]
PNDTT‐F3N–F	NT812:PC_71_BM	0.73	19.19	0.69	9.71	[122]
PNDIT‐F3N–Cl	NT812:PC_71_BM	0.73	19.53	0.70	10.26	[122]
PNDIT‐F3N–BIm4	NT812:PC_71_BM	0.73	19.56	0.71	10.23	[122]
PNDIT‐F3N–BArF4	NT812:PC_71_BM	0.53	17.80	0.45	4.49	[122]
NDIN‐Br	PTB7‐Th/PC_71_BM	0.78	17.40	0.73	9.88	[124]
NDIN‐Ac	PTB7‐Th/PC_71_BM	0.77	15.79	0.72	8.80	[124]
PDIN‐Br	PTB7‐Th/PC_71_BM	0.78	17.50	0.72	9.83	[124]
PDIN‐Ac	PTB7‐Th/PC_71_BM	0.77	16.47	0.73	9.25	[124]
P2G	PM6:Y6	0.85	25.20	0.74	16.00	[125]
PPDI‐F	PTB7‐Th/PC_71_BM	0.78	16.26	0.68	8.71	[126]
PPDI‐Cl	PTB7‐Th/PC_71_BM	0.78	15.98	0.72	9.03	[126]
PPDI‐OH	PTB7‐Th/PC_71_BM	0.78	16.23	0.73	9.27	[126]
PPDI‐Ac	PffBT4T‐2OD:PC_71_BM	0.76	17.27	0.71	9.40	[126]
PDI‐ionene	PBDTT‐TT:PC_71_BM	0.79	18.02	0.71	10.64	[127]
PDI‐PZ	PBDTT‐TT:PC_71_BM	0.63	18.49	0.60	7.34	[128]
C_60_‐PZ	(PBDTT‐TT:PC_71_BM	0.78	18.82	0.71	10.74	[128]
NDI‐NI	Y6: PM6: PC_71_BM	0.86	25.40	0.76	16.86	[129]
NDI‐CI	PTB7‐Th: PC_71_BM	0.77	18.48	0.58	8.52	[129]

### 3.3 Non‐Conjugated Polymers (NCPs)

**Figure 8:**
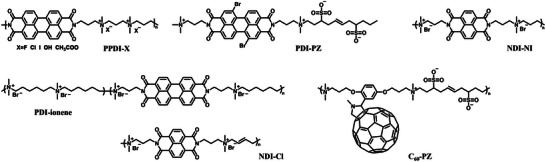
Chemical structures of non‐conjugated polymers as CILs.

Non‐conjugated polymer CIL materials usually contain π‐conjugated units that are covalently linked by non‐conjugated backbones. NCPs possess excellent film‐forming ability, which is the typical advantage for polymer materials, while exhibit high optical transparency. In particular, the functional units with large *π*‐conjugation framework may induce compact interchain π‐π stacking, which greatly promote the electron transport of NCP films. As a result, NCPs combines the good optical transmittance of organic small molecule, excellent charge transport property of CPs, and outstanding film‐forming ability of polymers, making them superior to other kinds of semiconductors in serving as CILs. To realize the high water/alcohol solubility and suitable energy levels for electron extraction via structure modification and chemical synthesis is an effective approach to improve the performance of NCP‐based CIL materials. **Figure** **8** presents the molecular structures of several commonly used NCP‐based CILs.

Based on the PDI unit, Huang et al.^[^ ^126^ ^]^ synthesized a series of *n*‐type polyelectrolytes modified by quaternary ammonium group with different counterions such as F^−^, Cl^−^, Br^−^, I^−^, OH^−^, and CH3COO^−^, via a simple quaternization polymerization/ion‐exchanging process. The polyelectrolytes possess good water/alcohol solubility and charge transport properties, solubility, which allows the multilayer depositions in the fabrication of OSCs. The incorporated PDI units and the self‐doping effect endow the polyelectrolytes with high electron mobility of ≈4.76 × 10^−4^ cm^2^ V^−1^ s^−1^, so the CILs exhibit excellent thickness‐insensitive performance in OSCs. Taking PPDI‐Ac as an example, the OSCs with 20 and 50 nm PPDI‐Ac as CILs show PCEs of 9.63% and 8.53%, respectively, which are higher than those of reference devices.

Liu and co‐workers make noticeable contribution in developing PDI‐ and NDI‐based NCPs as efficient CIL materials for OSCs. In 2018, Liu et al.^[^ ^127^ ^]^ demonstrated a novel strategy to prepare self‐doped PDI‐based ionene polymers, in which the semiconductor PDI components are embedded together with electrolyte dopants in the polymer backbone. By manipulating the ratio of PDI to ionene, the film morphology of CIL can be controlled, which helps to realize improved self‐doping and conductivity of the CIL. The polymer with high PDI content has good tolerance to the variation of interlayer thickness. PCE of 10.6% can be achieved in the OSC with the PDI‐based ionene CIL. The same group integrated PDI and C_60_ units into polymer zwitterions PDI‐PZ and C_60_‐PZ through the Menschuktin reaction via nucleophilic ring‐opening of a bis‐sultone 3,3′‐(but‐2‐ene‐1,4‐diyl)bis(1,2‐oxathiolane 2,2‐dioxide) with the PDI and C_60_ monomers.^[^ ^128^ ^]^ The electron mobility of C_60_‐PZ is more than two orders of magnitude higher than that of PDI‐PZ, reaching 8.85×10^−5^ cm^2^ V^−1^ s^−1^ . Using PBDTT‐TT:PC_71_BM as the active layer, OSCs with PDI‐PZ and C_60_‐PZ show PCEs of 7.37% and 10.74%, respectively. Liu et al.^[^ ^129^ ^]^ also integrated the electron‐deficient NDI unit into the ionene polymer to synthesize NDI‐NI and NDI‐CI with self‐doping properties. The cationic NCPs are a unique class of polyelectrolyte where the charged moieties are positioned within the polymer backbone rather than as pendants, so the NCPs have higher ionic density than other kinds of ionic polymers. Consequently, both the two polymers NDI‐NI and NDI‐CI exhibit excellent film‐forming property. It is worth noting that the two NCPs are universal CILs that afford fullerene‐based, non‐fullerene‐based, and ternary OSCs to show high PCEs over a wide range of CIL thicknesses (8‐37 nm), with a maximum PCE of 16.9%.

### 3.4 Transition Metal Oxides (TMOs)

TMOs are high‐performance semiconductors with unique superiority in charge transport. The TMOs have been extensively studied as electrode interlayer materials and have significantly improved the photoelectric conversion efficiency in optoelectronic devices. However, the intrinsic surface defects and the poor contact at the inorganic/organic interface between TMO and organic layer limit the charge extraction and transport efficiencies. In addition, the poor film‐forming property of TMOs usually leads to coarse film surface, which depresses the device performance. Thus, most TMOs have to be used with necessary modification. In recent years, many effective methods have been developed to optimize the optoelectronic and physical properties TMOs, which significantly improves the TMO performances for applications as CILs in efficient OSCs.(**Table** **3**).

**Table 3:** Devices parameters of OSCs with TMOs.

CILs	Photoactive Layer	*V*_oc_ [V]	*J*_sc_ [mA cm^−2^]	FF	PCE [%]	Ref.
ZnO:PBI‐H	PTB7‐Th:PC_71_BM	0.82	17.5	0.72	10.59	[131]
ZnO:PBI‐H	PTB7‐Th:PC71BM:DPPEZnP‐THE	0.77	18.7	0.74	11.03	[132]
ZnO:PBI‐Py	FBT‐Th4(1,4):PC_71_BM	0.77	18.3	0.75	10.80	[133]
ZnO:HO‐PBI‐iC7	PBDB‐T‐2F:Y6	0.83	25.3	0.74	15.95	[134]
ZnO:PEI	PTB7‐Th:PC_61_BM	0.81	16.0	0.63	8.16	[135]
ZnO:PASP	PM6:Y7	0.86	25.6	0.75	16.60	[136]
ZnO:PA	PM6: Y6	0.85	25.6	0.74	16.22	[137]
ZnO:Open‐C_60_	PTB7‐Th:PC_71_BM	0.72	17.4	0.66	8.26	[138]
ZnO: Open‐PCBM	PTB7‐Th:PC_71_BM	0.81	17.3	0.74	10.30	[138]
ZnO: Close‐C60	PTB7‐Th:PC_71_BM	0.77	17.1	0.65	8.58	[138]
ZnO: PFS‐FTEG	PTB7‐Th:PC_71_BM	0.80	18.3	0.72	10.60	[139]
ZnO: PFS‐FC	PTB7‐Th:PC_71_BM	0.80	18.0	0.69	10.30	[139]
ZnO: BA	PBDB‐TF:HDO‐4Cl:BTP‐eC9	0.86	26.9	0.79	18.40	[140]
ZnO: PA	BTP‐eC9	0.84	26.4	0.75	16.71	[141]
SnO_2_:CPTA	{en}FASnI3	0.69	16.5	0.65	7.40	[143]
SnO_2_:PAS	PM6:BTP‐eC9	0.84	26.4	0.77	17.12	[145]
SnO_2_:1‐DPAQ	PM6:PB2F:BTP‐eC9	0.86	26.5	0.79	18.10	[146]

#### 3.4.1 Zinc Oxide (ZnO)s

Owing to outstanding optoelectronic properties and simple preparation, ZnO is predominantly used as the n‐type CIL material in OSCs. The excellent overall performance of ZnO in serving as CILs is derived from its suitable energy level for electron extraction, high electron mobility, and high transparency through the whole visible region. The early researches of ZnO are mainly focused on ZnO nanoparticles including preparation, processing, and modification. However, ZnO nanoparticles usually result in coarse film surfaces, resulting in serious current leakage in devices. In recent years, sol‐gel process was widely employed for the preparation of ZnO films. The ZnO films prepared by the sol‐gel method not only exhibit smooth and uniform surface, but also possess suitable optoelectronic properties for electron collection. At present, the sol‐gel method for preparing ZnO has been greatly optimized, which significantly improves the ZnO performances in cathode modifications.

**Figure 9:**
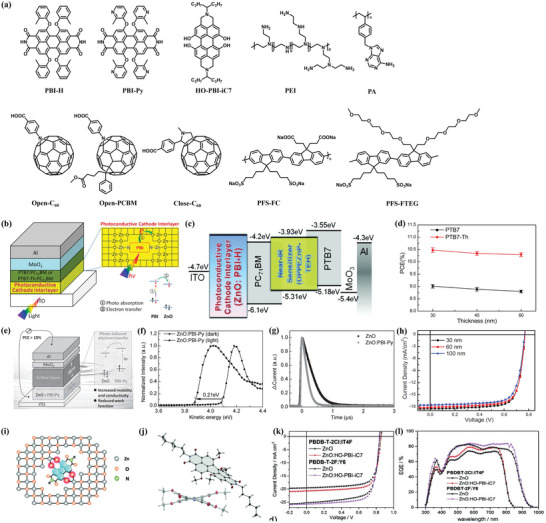
a) The representative molecules for modifying ZnO in the fabrication of OSCs. b) ZnO:PBI‐H device and its electron transfer schematic diagram. c) Energy level diagram of ZnO:PBI‐H device. Reproduced with permission.^[^ ^132^ ^]^ Copyright 2016, Wiley‐VCH. d) Effects of the thickness of the ZnO:PBI‐H cathode interlayer on device performance. Reproduced with permission.^[^ ^131^ ^]^ Copyright 2015, American Chemical Society. e) Schematic illustration of the working mechanism of the ZnO: PBI‐Py device. f) UPS spectra of ZnO:PBI‐Py on the ITO substrates in dark and under white light illumination. g) Transient photocurrent as a function of time for the iPSCs in TPC measurement. h) *J*–*V* characteristics of iPSCs with different thicknesses of ZnO: PBI‐Py. Reproduced with permission.^[^ ^133^ ^]^ Copyright 2016, Wiley‐VCH. i) Schematic illustration of HO‐PBI dyes incorporated into the ZnO wurtzite lattice by O‐Zn bonding. j) Solid‐state structure of MeO‐PBI‐C6 and the view of the molecule along the long axis of the PBI core. k) *J*–*V* characteristics of ZnO: HO‐PBI. l) EQE spectra of the devices based on PBDBT‐2Cl:IT4F and PBDB‐T‐2F:Y6 employing ZnO or ZnO:HO‐PBI‐iC7 as the cathode interlayer. Reproduced with permission.^[^ ^134^ ^]^ Copyright 2019, Wiley‐VCH.

The *n*‐doping methods have been proved to be effective approaches to significantly enhance the electric conductivity of ZnO and passivate the surface defects. Many organic compounds were developed as dopants to modify the surface of ZnO, which greatly improve the optoelectronic properties of the ZnO‐based CIL materials. Xie et al.^[^ ^130^, ^131^ ^]^ designed and synthesized a light absorber PBI‐H and obtained a highly photoconductive sol‐gel‐derived ZnO CIL by doping a very small amount of PBI‐H.The PBI‐H molecule may form a N‐Zn bond with ZnO during the thermal treatment, which not only produces high conductivity of 4.5 × 10^−3^ S/m for the ZnO:PBI‐H film, but also enhances the interfacial bonding and promotes electron extraction from the active layer are displayed in **Figure** **9**b,c. Based on PTB7‐Th:PC_71_BM active layer, the ZnO:PBI‐H‐modified OSC shows a PCE of 10.5% (Figure 9d). Afterward, the same group synthesized a deep absorbing porphyrin small molecule DPPEZnP‐THE as near‐IR sensitization to develop a ternary composite PTB7‐Th:PC_71_BM:DPPEZnP‐THE showing absorption beyond 900 nm.^[^ ^132^ ^]^ By using the ternary composite as active layer, a high PCE of 11.03% was achieved in the OSC with the ZnO:PBI‐H CIL. The above results suggest that the doping of ZnO by organic dyes to form organic‐inorganic composite layer is effective for developing new CILs. By using pyridine to substitute the benzene pendants in PBI‐H, Xie and co‐workers developed using a mild acid water‐soluble PBI‐Py as the dopant to construct the ZnO:PBI‐Py film(Figure 9e), which showed increased electron mobility and reduced WF under illumination (Figure 9f).^[^ ^133^ ^]^ The added acetic acid can convert PBI‐Py into cationic pyridinium derivative, which facilitates the formation of monodispersed PBI‐Py in ZnO matrix. Based on a 300‐nm thick blend film FBT‐Th_4_(1,4):PC_71_BM as active layer, the ZnO:PBI‐Py‐modified OSC exhibited a PCE of 10.8%(Figure 9g). As shown in Figure 9h, the photovoltaic efficiency could be maintained higher than 10% with ZnO:PBI‐Py thickness variations from 30 to 100 nm. To achieve an even dispersion of organic dopant in ZnO, Xie introduced four hydroxy (HO) groups into the bay areas of PDI unit to develop a new HO‐PBI ligand that can complex Zn^2+^ ions and photosensitize the ZnO thin films.^[^ ^134^ ^]^ It is suggested that the stable coordination between Zn^2+^ ions and PBI‐Py favors the dispersion of the PBI‐Py photosensitizer evenly into metal oxide films to fabricate organic‐inorganic hybrid CILs for OSCs (Figure 9i,j). A remarkable PCE of 15.95% was achieved in OSCs by the incorporation of the ZnO:HO‐PBI hybrid interlayers is exhibited in Figure 9k,l.

Nonconjugated polymer can also be used to modify ZnO nanoparticles. Ma et al.^[^ ^135^ ^]^ employed the commercially available polyethylenimine (PEI) to modify the ZnO colloidal particles. The cooperation of PEI in nano ZnO offers a good film‐forming ability of the composite material, which is beneficial for device fabrication. The PCE of the PTB7‐Th:PC_61_BM devices reached to 8.16%, and showed no obvious photovoltaic performance loss with the increase of the ZnO:PEI thickness to 60 nm. It is found that many biopolymers with electron‐donating ability can *n*‐doping ZnO, improving the optoelectronic properties and optimizing the surface energy of ZnO‐based CILs. Zhou et al.^[^ ^136^ ^]^ demonstrate an environmentally friendly non‐conjugated biopolymer polyaspartic acid (PASP) as interfacial modifier to modify the sol‐gel‐prepared ZnO for fabricating inverted OSCs. An interfacial doping effect in ZnO can be induced by modifying with PASP, which plays a key role in improving the charge extraction of the CIL. The PCE of OSCs based on the PM6:Y7 active layer was boosted from 15.7% to 16.6% and could be maintained by 86% of its original value after 25 days in a nitrogen environment. Liu and co‐workers utilized the reversible addition‐fragmentation chain transfer (RAFT) polymerization to integrate nucleobase adenine into a non‐conjugated polymer as pendent groups to synthesize an adenine‐based polymer (PA) for modifying ZnO nanoparticle.^[^ ^137^ ^]^ PA efficiently passivates the defects, increases the conductivity, and decreases the work function of the ZnO films, so the modified ZnO can work well in a wide thickness range from 18 to 100 nm. Devices containing PA‐modified ZnO CIL yielded a maximum photovoltaic efficiency of 16.2%.

Li and co‐workers reported a series of aza[60]fulleroid derivatives Open‐C_60_, Open‐PCBM, and Close‐C_60_ as interfacial modifiers to passivate the surface defects of ZnO.^[^ ^138^ ^]^ By facilitating effective *π*‐conjugation between fullerene and aromatic addends, the electronic coupling between fullerene and the underneath TMOs (through conjugated anchors) could be enhanced, leading to an improvement of electron collection ability for ZnO CILs. The interfacial modifiers can also change the surface energy of ZnO, which optimizes the vertical composition distribution of the BHJ active layer to facilitate charge transport. A PCE enhancement from 9.51% to 10.3% was achieved by the incorporation of the Open‐PCBM modifier. Wong et al.^[^ ^139^ ^]^ synthesized two fluorene‐based conjugated polyelectrolytes PFS‐FTEG and PFS‐FC to act as buffer layers for modifying ZnO. The PFS‐FTEG‐modified ZnO exhibited high transparency and smooth CIL, and the corresponding OSC showed a PCE of 10.6%, which is much higher than that of the device with bare ZnO (PCE = 9.5%).

Zheng and co‐workers have made great effort to improve the optoelectronic and surface properties of sol‐gel‐prepared ZnO CILs, by which the significant enhancements in OSC efficiencies were achieved. Zheng et al.^[^ ^140^ ^]^ found that the removal of the residual amine that was added in the sol‐gel preparation of ZnO could effectively prevent the reaction between amine and organic electron acceptor, which significantly enhance the OSC efficiencies. Through rational screening, boric acid (BA) is selected as the amine‐removing agent because of its suitable acidic dissociation constant. Owing to the proper *n*‐doping of ZnO by the removal of residual amine, the WF of the ZnO film was decreased from 3.86 to 3.79 eV, leading to a 12 MeV *V*_oc_ increase in OSCs. By using a ternary blend PBDB‐TF:HDO‐4Cl:BTP‐eC9 as active layer, the 0.04 and 1.00 cm^2^ OSCs based on the BA‐rinsed ZnO CILs exhibited PCEs of 18.40% and 17.42%, respectively. Based on the above results, Zheng further demonstrated a printable ZnO CIL that can be processed by meniscus‐guided coating with the promotion of sol‐gel technology.^[^ ^141^ ^]^ By adopting a proper Lewis base PA in the sol‐gel method, the Marangoni recirculation in meniscus and the annealing temperature of the sol‐gel ZnO precursor are effectively modulated, and consequently the blade‐coated ZnO can exhibit low WF, high conductivity, and smooth surface. In particular, a lower annealing temperature of 130 °C is needed to convert the PA‐based gel into ZnO, which makes PA‐ZnO an ideal candidate to be used in the fabrication of flexible OSCs. As a result, the 1.00 cm^2^ flexible OSC with the PA‐ZnO CIL was fabricated, exhibiting good photostability and an outstanding PCE of 16.71%, which is the best value among the 1.00 cm^2^ flexible OSCs.

#### 3.4.2 Tin Oxide (SnO_2_)

It is well known that the poor device stability of the inverted nonfullerene (NF) organic solar cells (OSCs) under solar light illumination have become a critical problem in the development of commercial OSCs. At present, zinc oxide (ZnO) as the electron transporting layer (CIL) is generally used in the NF OSCs, but its photocatalytic activity under UV illumination can cause a decomposition of photoactive layer; this is certainly to the detriment of the operational stability of the ZnO‐based NF OSCs. Zhou et al. demonstrated that ZnO with photocatalytic activity could induce the decomposition of the NF acceptor under UV illumination, and the use of SnO_2_ as CIL to replace ZnO exhibited higher efficiency and better operational stability than the ZnO‐based NF OSC.^[^ ^142^ ^]^ When the nonfullerene acceptors IT‐4F films were used as photoactive layer, the C═C linkage in IT‐4F was disrupted under UV illumination by the photocatalytic effect of ZnO. However, SnO_2_ as CIL could reduce the photocatalytic effect because SnO_2_ of wider bandgap did not absorb photons in the AM1.5 irradiation spectrum, so that SnO_2_ showed superior illumination stability and PCE to ZnO.

Although the PCE of Pb‐based hybrid perovskite solar cells (PSCs) still remain a remarkable success, the toxicity of lead hinders the progress in application of PSCs. Thus Sn‐based hybrid perovskite solar cells have recently attracted much interest. Liang et al.^[^ ^143^ ^]^ reported a highly efficient Sn‐based hybrid perovskite solar cell using SnO_2_ as CIL, so that PCE of 7.40% and *V*_oc_ of 0.72 V are achieved for FASnI_3_ solar cell with a n–i–p planar architecture. The hydrophilic derivative of fullerene C_60_ pyrrolidine tris‐acid (CPTA) was deposited between SnO_2_ and the perovskite to prevent the oxidation process of Sn^2+^ to Sn^4+^ from doping the perovskites. Also, the introduction of CPTA is very important for improving electron transport performance. The research results from Liang et al. indicated that the Sn‐based hybrid PSCs with only‐SnO_2_ as CIL showed very low efficiency, but the solar cells with SnO_2_‐CPTA electron transport layer could obtain a high PCE of 6.54%.

It is well known that the mass production of OSCs has to require printable electrode interlayer along with good thickness tolerance, and thus the development of printable electrode interlayers with good thickness tolerance is of considerable significance. Tan et al.^[^ ^144^ ^]^ developed a facile room‐temperature process to prepare SnO_2_ films as CIL of OSCs. The SnO_2_ films are prepared by spin‐coating or blade‐coating the chemical‐precipitated SnO_2_ colloid precursor. Encouragingly, the SnO_2_ films as cathode interlayers of OSCs not only possess outstanding optical, electrical, and charge transport property, but also exhibit excellent thickness‐insensitivity. By using 10‐nm spin‐coated SnO_2_ as CIL, the high PCE of 16.10% is achieved for the PM6:Y6‐based OSCs. In particular, the PCE of 13.07% can remain when the thickness of the SnO_2_ film is up to 160 nm.

Although SnO_2_ has been extensively used as CIL in OSCs due to its ultrahigh transparency, high electrical conductivity, and excellent chemical stability, the presence of the surface hydroxyl groups and the defects of oxygen vacancy on SnO_2_ can cause charge recombination. Recently, Tan et al.^[^ ^145^ ^]^ demonstrated that the introduction of an electrolyte molecule 4‐(dimethyl(pyridin‐2‐yl) ammonio) butane‐1‐sulfonate (PAS) into SnO_2_ films could evidently improve the performance of the inverted OSCs, because of the improved conductivity of the SnO_2_ layer. Moreover, the PAS could enhance the interface connection between SnO_2_ and the active layer, and the PAS‐doped SnO_2_ could boost charge transport so as to increase electron conductivity. By using PM6:Y6 as active layer and pristine SnO_2_ as CIL, the inverted OSC attained the PCE of 14.72%, while the device using PAS‐doped SnO_2_ as CIL could achieve a PCE of 16.37%. In the same research group, a new strategy of interface modification by means of multi‐site coordination was carried out for optimizing the performance of SnO_2_ CIL.^[^ ^146^ ^]^ They introduce an anthraquinone derivative (1‐DPAQ) to passivate the surface defects of SnO_2_ CIL, so that the work function can be optimized and the electron conductivity of the SnO_2_ is improved obviously. The existence of coordination interactions between SnO_2_ and 1‐DPAQ can fill the oxygen vacancy of SnO_2_, so as to restrain the charge recombination and improve the charge collection ability.

#### 3.4.3 Titanium Oxide (TiO_2_)

In the development of OSCs, researchers are deeply convinced that charge transport layers, including HTL and CIL, play an important role in the performance enhancement of OSCs. Therefore, recent research on charge transport layer materials has obtained some interesting findings. For the CIL, although metal Ca of low work function is generally selected for optimizing the electrical contact, the solution‐processed metal oxides such as ZnO or TiO*x* are also used widely. Hadipour et al.^[^ ^147^ ^]^ systematically investigated the performance enhancement of the OSCs based on P3HT:PCBM with various charge transport layers, and demonstrated that the optical losses (refractive index) obviously influence the performance of OSCs. By using a low refractive index TiOx as CIL replacing metal Ca in the inverted OSC device, the parasitic absorption losses can considerably reduce, so that the *J*_sc_ increases by 25%; this is because the use of the non‐absorbing TiO_x_ as CIL can lead to an increase in EQE at λ > 450 nm. Hou et al.^[^ ^74^ ^]^ find an advanced material of interconnecting layer, namely electron beam evaporated TiOx and PEDOT:PSS, for tandem organic solar cell. The deposited TiOx/PEDOT:PSS interface is flat, uniform, dense, and acid‐resisting, which is favorable to form a smooth and dense PEDOT:PSS film. The TiO_1.62_, TiO_1.76_, and TiO_1.89_ were prepared through the controlled O_2_ fluxes at 0.1, 0.3, and 0.5 SCCM, respectively. Due to the neat interface, high conductivity, suitable energy levels, and low Schottky barrier of the TiO_1.76_/PEDOT:PSS layer, a PCE of 20.27% can be achieved for the corresponding tandem OSC.

Interestingly, a titanium coordination compound with organic ligand could also be employed as CIL in the inverted OSC, because of the weak points that TiOx was prepared ether by vacuum‐evaporation or with high‐temperature treatment. Li et al.^[^ ^148^ ^]^ reported the use of an alcohol‐soluble chelate coordination compound titanium diisopropoxide bis(2,4‐pentanedionate) (TIPD) as CIL to improve the performance of the inverted OSC, in which the CIL was prepared by spin‐coating the TIPD isopropanol solution on ITO electrode and then thermal treated at 60–170 °C for 10–30 min. The advantages of using TIPD as CIL are its solution processability, lower annealing temperature, low cost, and high transparency for the visible light. The PCE of the inverted OSC with TIPD CIL could reach 7.4%, while the PCE of the conventional OSC without TIPD exhibited 6.4%. The authors think that the PCE increased by 16% is benefitted from a surface transformation of TIPD CIL from hydrophilic to hydrophobic, which is favorable to the active layer formation.

## 4 Conclusions and Outlook

The CILs play an important role in achieving high photovoltaic efficiency and good long‐term stability in OSCs. Benefitting from the excellent charge transporting property, the SPS‐based CILs show obvious superiorities to their counterparts constructed based on the interfacial dipole mechanism. Particularly, most SPS‐based CILs exhibit good compatibility with large‐area processing technologies, which is crucial for the practical use. The material characteristics, molecular design strategies, advantages, and existing problems of SPS‐based CIL materials are discussed. Afterward, we reviewed a wide range of SPS materials that have been explored as CILs including organic small molecules, conjugated polymers, non‐conjugated polymers, and TMOs. All the current SPS‐based CILs show great potential but none of them is proved to be the perfect choice since each has its own advantages and disadvantages. In order to better promote the progress of SPS materials and their applications in OSCs, we have identified several key CIL‐related issues that require further investigation. These areas include:

1) Development of SPS‐based CILs for constructing interconnecting layers for tandem solar cells

The construction of tandem OSCs with multijunction architecture is an effective approach to solve to problem of the thermal exciton relaxation, providing a promising solution to improving the PCE. Since tandem OSCs are composed of multiple sub‐cells, the optimization of interfacial properties becomes even more important. The CILs for tandem OSCs need to have adequate solvent resistance to facilitate the solution processing of multilayer cells. Moreover, the CILs should possess good wettability on various substrates such as active layer and hole transporting layer. To ensure appropriate current matching, the optoelectronic properties of CILs must match well with those of hole‐transporting layers, so an efficient charge recombination can occur in the specially designed p‐n junction interconnecting layers.

2) SPS‐based CILs for flexible transparent electrode

One significant advantage of organic photovoltaic technology is to fabricate flexible device, in which flexible transparent electrodes such as conducting polymers, carbon nanotubes, and metal nanowires are used. However, the applications of most solution‐processed CILs in flexible OSCs are seldom explored. An in‐depth understanding of the interfacial properties between the SPS‐based CILs and flexible transparent electrodes is strongly needed. To improve the bending resistance of flexible OSCs, new CILs with high mechanical strength and improved adhesion with flexible electrodes are strongly desired.

3) Printable CILs for fabricating large‐area OSCs

To realize the commercialization of OSCs, the large‐scale manufacturing of large‐area devices must be achieved. In industrial productions, OSCs will be fabricated through printing technologies, such as gravure printing, screen printing, and R2R printing. Although several CILs have been demonstrated to be processed by printing methods, few CIL material is reported to be successfully used in fabricating efficient OSCs with device area of over 10 cm^2^. The conductivity of SPS‐based CILs needs to be enhanced to facilitate electron transport, which may improve the thickness insensitivity of SPS‐based CILs. Particularly, in fabricating all‐printed OSCs, the interfaces in the devices formed between TMOs, organic layers, and electrodes all have very different optoelectronic and wetting properties. Therefore, the development of SPS‐based materials that match various functional layers is crucial for all‐printed OSCs. Besides the development of new materials, the optimization of processing technologies is also strongly needed. The kinetics of film printing should be systematically investigated and the related theoretical model is desired for preparing defect‐free CILs.

## Conflict of Interest

The authors declare no conflict of interest.
